# Association of probiotic use with nivolumab effectiveness against various cancers: A multicenter retrospective cohort study

**DOI:** 10.1002/cam4.6313

**Published:** 2023-07-08

**Authors:** Junya Arai, Ryota Niikura, Yoku Hayakawa, Nobumi Suzuki, Tetsuro Honda, Takuma Okamura, Kenkei Hasatani, Naohiro Yoshida, Tsutomu Nishida, Tetsuya Sumiyoshi, Shu Kiyotoki, Takashi Ikeya, Masahiro Arai, Narikazu Boku, Mitsuhiro Fujishiro

**Affiliations:** ^1^ Department of Gastroenterology, Graduate School of Medicine The University of Tokyo Tokyo Japan; ^2^ Department of Gastroenterology The Institute for Medical Science, Asahi Life Foundation Tokyo Japan; ^3^ Gastroenterological Endoscopy Tokyo Medical University Tokyo Japan; ^4^ Department of Gastroenterology Nagasaki Harbor Medical Center Nagasaki Japan; ^5^ Department of Gastroenterology Fukui Prefectural Hospital Fukui Japan; ^6^ Department of Gastroenterology Ishikawa Prefectural Central Hospital Kanazawa‐shi Ishikawa Japan; ^7^ Department of Gastroenterology Toyonaka Municipal Hospital Osaka Japan; ^8^ Department of Gastroenterology Tonan Hospital Sapporo‐shi Hokkaido Japan; ^9^ Department of Gastroenterology Shuto General Hospital Yamaguchi Japan; ^10^ Department of Gastroenterology St. Luke's International Hospital Tokyo Japan; ^11^ Department of Gastroenterology Nerima Hikarigaoka Hospital Tokyo Japan

**Keywords:** CBM588, gastric cancer, nivolumab, probiotics

## Abstract

**Background:**

Previous studies have revealed an association between probiotic use and effectiveness of immune checkpoint inhibitors in renal and lung cancers. However, little is known regarding other cancers, including gastrointestinal cancer.

**Methods:**

To address this issue, we conducted a multicenter retrospective cohort study and the duration of nivolumab treatment for various cancers was compared between probiotic users and non‐users.

**Results and Conclusions:**

In total, 488 patients who received nivolumab therapy were included. In all cancers, no significant differences in treatment duration of nivolumab were observed between probiotic users and non‐users (median 62.0 vs. 56.0, hazard ratio = 1.02, *p* = 0.825), whereas probiotic use, compared with non‐use, in patients with gastric cancer was significantly associated with a longer duration of nivolumab treatment (55.0 vs. 31.0 days, hazard ratio = 0.69, *p* = 0.039). In conclusion, probiotics may improve the response to nivolumab and potentially prolong progression‐free survival in patients with gastric cancer.

## Graphical abstract

Previous studies have revealed an association between probiotic use and effectiveness of immune checkpoint inhibitors in renal and lung cancers. This study showed that probiotics may improve the response to nivolumab and potentially prolong progression‐free survival in patients with gastric cancer.

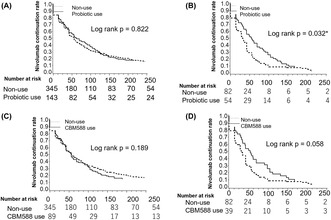

## 1 INTRODUCTION

Immune checkpoint inhibitors (ICIs) are now widely used for the treatment of various solid tumors, and several previous studies have revealed an association between their effectiveness and the gut microbiota.^1^, ^2^, ^3^, ^4^ Moreover, probiotic use is now discussed in relation to the efficacy of ICIs.^5^, ^6^ According to a recent report, a randomized phase 1 trial was conducted to evaluate the effects of CBM588, a bifidogenic probiotic product, in patients with metastatic renal cell carcinoma treated with nivolumab plus ipilimumab. Patients receiving nivolumab plus ipilimumab with CBM588 showed significantly longer progression‐free survival (PFS) than those without CBM588 (12.7 months versus 2.5 months, hazard ratio 0.15, 95% confidence interval 0.05–0.47, *p* = 0.001), suggesting the potential benefits of probiotics in combination with immune checkpoint inhibitors.^5^ Moreover, a retrospective study from Japan showed similar effects of CBM588, as patients with non‐small‐cell lung carcinoma receiving ICIs plus CBM588 showed longer PFS and overall survival (OS) than those not receiving CBM588.^6^ However, as various types of probiotics other than CBM‐588 are commonly used in Japan, we hypothesized that these probiotics could have similar effects on other solid tumors, including lung, esophageal, colorectal, and gastric cancers.

To test this hypothesis, we performed a multicenter retrospective cohort study to evaluate the association between the effectiveness of nivolumab treatment for various solid tumors and the use of probiotics, including the bifidogenic probiotics CBM588 and *Lactobacillus*, and *Bifidobacterium*.

## 2 MATERIALS AND METHODS

### 2.1 Study design, setting, and patients

This retrospective cohort study was performed using clinical practice‐based databases from nine hospitals including Tonan Hospital, the University of Tokyo Hospital, Shuto General Hospital, Fukui Prefectural Hospital, Nerima Hikarigaoka Hospital, St. Luke's International Hospital, Toyonaka Municipal Hospital, Ishikawa Prefectural Central Hospital, and Nagasaki Minato Medical Center. The study period was from April 2014 to March 2019. This study was approved by the Institutional Review Board of the University of Tokyo Hospital (approval no. 2019161NI). Patient consent was waived because data are anonymous.

### 2.2 Outcomes and variables

The primary outcome was the continuation of nivolumab therapy. We used the treatment duration of nivolumab as a surrogate variable for efficacy and compared it between probiotic users and non‐users and between CBM588 users and non‐users given that the treatment duration of nivolumab is clinically relevant.^7^ During the follow‐up period, nivolumab was used as monotherapy in esophageal, stomach, colorectal and lung cancers. Only 8 patients with kidney cancer and 12 patients with the other cancers were treated with combination therapy of Nivolumab and Ipilimumab.

Probiotics are usually prescribed for the treatment or prevention of diarrhea. Probiotic medications included miya‐BM®, bio‐three®, bifisgen®, biofermin®, LAC‐B®, biosmin®, and lebenin®. These drugs are taken three times daily, and we defined the probiotic use as a prescription for >90 days. A total of 140 patients are prescribed probiotics one tablet at a time, whereas only 3 patients were two tablets at a time.

We also evaluated the following clinical factors: age, sex, and comorbidities. The following comorbidities (based on ICD‐10 codes) were included: atrial fibrillation, acquired immunodeficiency syndrome, arterial thrombosis, carotid disease, cerebrovascular disease, chronic heart failure, chronic kidney disease (stage 5), dementia, diabetes mellitus with or without complications, deep vein thrombosis, hemiplegia, dyslipidemia, ischemic heart disease, liver disorder (mild/severe), malignancy with or without metastasis, peripheral vascular disease, pulmonary disease, rheumatic disease, transient ischemic attack, peptic ulcer disease, unstable angina, and valvular disease. The Charlson comorbidity index was calculated using these data.^8^

### 2.3 Statistical analysis

Cox hazard models were used to estimate hazard ratios (HRs) and 95% confidence intervals (Cis) for nivolumab continuation. The Kaplan–Meier method was used to estimate the treatment duration of nivolumab until 250 days. Statistical significance was set at *p* < 0.05. All statistical analyses were performed using the SAS software (ver. 9.4; SAS Institute, Cary, NC, USA).

## 3 RESULTS

### 3.1 Patient characteristics

A total of 488 patients who received nivolumab were selected from our database. The primary tumor organs were the esophagus (32.8%), stomach (27.87%), colorectum (1.84%), kidneys (13.11%), lungs (41.80%), and others (12.09%). Of these, 29.30% of the patients were administered with probiotics. Patient characteristics are shown in Table S1 and S2.

### 3.2 Association between probiotic use and duration of nivolumab treatment for all cancers

**FIGURE 1:**
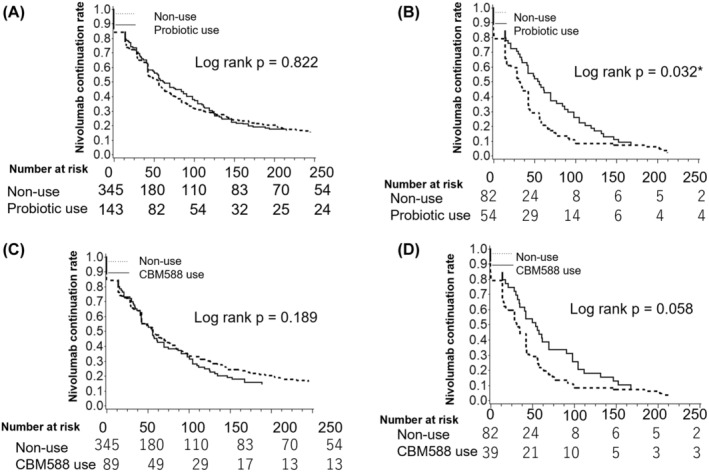
Cumulative incidence of nivolumab discontinuation in probiotic users versus non‐users for (A) the total cohort and (B) gastric cancer and CBM588 users versus non‐users for (C) the total cohort and (D) gastric cancer. *Shows <0.05.

No significant differences in a treatment duration of nivolumab were observed between probiotics users (including CBM588) and non‐users (median 62.0 vs. 56.0, HR = 1.02, 95% CI 0.84–1.24, *p* = 0.825) in all cancers (Table 1). The cumulative incidences of nivolumab continuation in all cancers were 37.76% and 32.58% at 100 days and 17.78% and 14.61% at 200 days in the probiotics and CBM588 groups, respectively, compared with 31.59% at 100 days and 20.29% at 200 days in patients not using probiotics (Figure 1A, *p* = 0.822 and Figure 1C, *p* = 0.189). Moreover, probiotic use was not significantly associated with nivolumab use duration for any other cancers except for gastric cancer (Table 1 and Figure S1).

**TABLE 1:** Treatment duration of nivolumab according to probiotics use for several cancers.

Organ of the primary tumor	Any probiotic user (*N* = 143)	Probiotic non‐user (*N* = 345)	HR (95% CI)	*p*
All	62.0	56.0	1.02 (0.84–1.24)	0.825
Esophagus (*n* = 16)	42.0	56.0	0.78 (0.25–2.47)	0.672
Stomach (*n* = 136)	55.0	31.0	0.69 (0.49–0.98)	0.039*
Colorectum (*n* = 9)	99.5	63.0	1.26 (0.27–5.81)	0.767
Kidney (*n* = 64)	42.0	89.0	1.67 (0.97–2.86)	0.063
Lung (*n* = 204)	105.0	77.0	0.97 (0.81–1.33)	0.848

Organ of the primary tumor	CBM588 user (*N* = 89)	Probiotic non‐user (*N* = 345)	HR (95% CI)	*p*
All	56.0	56.0	1.14 (0.90–1.44)	0.289
Esophagus (*n* = 15)	39.0	56.0	1.74 (0.52–5.84)	0.371
Stomach (*n* = 121)	54.0	31.0	0.70 (0.47–1.03)	0.068
Colorectum (*n* = 9)	99.5	63.0	1.26 (0.27–5.81)	0.767
Kidney (*n* = 56)	41.5	89.0	1.87 (0.99–3.51)	0.052
Lung (*n* = 183)	88.0	77.0	1.07 (0.73–1.57)	0.721

Abbreviations: CI, confidence interval; HR, hazard ratio.

* Shows <0.05.

### 3.3 Association between probiotic use and duration of nivolumab treatment for gastric cancers

Probiotic use, compared with non‐use, in patients with gastric cancer was significantly associated with a longer duration of nivolumab treatment (55.0 vs. 31.0 days, HR = 0.69, 95% CI 0.49–0.98, *p* = 0.039) (Table 1). The cumulative incidence of nivolumab continuation for gastric cancer was 25.93% and 25.64% at 100 days, and 7.41% and 7.69% at 200 days in patients using probiotics and CBM588, respectively, and 8.54% at 100 days and 6.10% at 200 days in patients not using probiotics (Figure 1B, *p =* 0.032 and Figure 1D, *p* = 0.058).

## 4 DISCUSSION

In this multicenter cohort study, we found that probiotic use was significantly associated with a longer duration of nivolumab treatment in patients with gastric cancer.

Nivolumab is now widely used as a first‐line regimen for gastric cancer, as it reportedly provides longer PFS and OS.^9^ The clinical efficacy of ICIs in gastric cancer is often discussed in relation to the gut microbiota similar to those of other cancers.^10^ Specific bacteria, such as *Bifidobacterium*, may modify the bacterial flora and alter the antitumor immune microenvironment, resulting in improved responses to nivolumab in gastric cancers.^11^, ^12^, ^13^, ^14^ These effects on the gut microbiota may vary among races, regions, cancer types, or cancer stages. Nevertheless, probiotics, such as CBM588 or other bifidogenic medications, may boost favorable immune reactions in a subset of cancer patients and can be clinically applied in the near future.

The solid mechanism for this impact is still unknown. However, previous studies have shown that patients with abundant levels of butyrate in their feces tend to have longer progression‐free survival (PFS) when treated with ICIs.^15^, ^16^ Probiotic use may increase the presence of butyrate‐producing bacteria, such as Clostridium butyricum. Additionally, a recent report suggests that the use of CBM588, a specific probiotic strain, can increase the production of chemokines, including CCL2 (MCP‐1), CCL4 (MIP‐1β), CXCL9 (MIG), and CXCL10. These chemokines play a role in stimulating cytotoxic T cells and T helper 1 cells, resulting in enhanced antitumor immunity.^5^ The exact mechanism by which probiotic use impacts ICI effectiveness requires further investigation.

In addition, our previous studies have suggested that bifidogenic probiotics decrease the risk of gastric cancer after *Helicobacter pylori* eradication or endoscopic resection.^17^, ^18^ Thus, probiotics may be helpful in cancer prevention, possibly by activating microbiota‐driven antitumor immunity. The exact mechanisms underlying the roles of the gut microbiota and probiotics and efficacy of ICIs remain to be elucidated in future research.

Probiotic use was not associated with nivolumab continuation rate for renal and lung cancers in contrast to previous reports. We speculate that this discrepancy may be due to the differences in patient backgrounds and therapeutic regimens. However, further studies are required to confirm this hypothesis.

Our study has several strengths. First, we simultaneously evaluated the association between probiotic use and ICIs' effectiveness in several solid tumors. Second, this study used a multicenter cohort data. Nevertheless, this study has several limitations. First, our study was a retrospective study. Second, detailed information on cancer profiles was limited to our database. Third, we also lacked information about the response for Nivolumab, PFS, and toxicity. Regarding OS, the result is similar to that of the primary outcome, although the mortality data are not accurate due to the database design (Table S3). Fourth, our sample size was small. Similar prospective studies using larger databases are required in the future.

In conclusion, we suggest that probiotics may improve response to nivolumab and potentially prolong PFS in gastric cancer.

## AUTHOR CONTRIBUTIONS

**Junya Arai:** Conceptualization (equal); data curation (equal); formal analysis (equal); investigation (equal); methodology (equal); writing – original draft (equal). **Ryota Niikura:** Conceptualization (equal); formal analysis (equal); writing – review and editing (equal). **Yoku Hayakawa:** Conceptualization (equal); formal analysis (equal); supervision (equal); writing – review and editing (equal). **Nobumi Suzuki:** Writing – review and editing (equal). **Tetsuo Honda:** Writing – review and editing (equal). **Takuma Okamura:** Writing – review and editing (equal). **Kenkei Hasatani:** Writing – review and editing (equal). **Naohiro Yoshida:** Writing – review and editing (equal). **Tsutomu Nishida:** Writing – review and editing (equal). **Tetsuya Sumiyoshi:** Writing – review and editing (equal). **Shu Kiyotoki:** Writing – review and editing (equal). **Takashi Ikeya:** Writing – review and editing (equal). **Masahiro Arai:** Writing – review and editing (equal). **Narikazu Boku:** Writing – review and editing (equal). **Mitsuhiro Fujishiro:** Writing – review and editing (equal).

## FUNDING INFORMATION

This study was supported by the KAKENHI Grant‐in‐Aid for Scientific Research (Grant Nos. 23H02744 [YH] and 23K07448 [RN]) and AMED (PRIME and P‐CREATE). The funding agencies had no role in the study design, data collection and analysis, decision to publish, or manuscript preparation.

## CONFLICT OF INTEREST STATEMENT

The authors declare no conflicts of interest.

## STATEMENT OF ETHICS

The study was conducted according to the guidelines of the Declaration of Helsinki and approved by the Institutional Review Board of the University of Tokyo (ID: 2058‐[2]).

## Supporting information


Data S1:

## Data Availability

The data that support the findings of this study are available from the corresponding author (YH and RN), upon reasonable request.
