# Supplementary material for: Association of probiotic use with nivolumab effectiveness against various cancers: A multicenter retrospective cohort study
Source: Cancer Med. 2023 Jul 8;12(16):16876–80. doi: 10.1002/cam4.6313 (PMC10501230; doi:10.1002/cam4.6313)

**Supplementary Table 1. Patient characteristics (number of patients [%] or mean** ± **standard deviation)**

| **Clinical characteristics** | **Overall cohort**  **(n = 488)** |
| --- | --- |
| Male sex | 361 (73.98) |
| Age (years) | 67.29 ± 10.60 |
| Charlson comorbidity index | 5.69 ± 3.65 |
| The duration of nivolumab administration | 130.27 ± 191.08 |
| The organ of the primary tumor |  |
| Esophagus | 16 (3.28) |
| Stomach | 136 (27.87) |
| Colorectum | 9 (1.84) |
| Kidney | 64 (13.11) |
| Lung | 204 (41.80) |
| Other | 59 (12.09) |
| Probiotics administration | 143 (29.30) |
| CBM588 | 89 (18.24) |

**Supplementary Table 2. The number of probiotic and CBM588 users with various tumors (number of patients [%])**

| **Organ of the primary tumor** | **Probiotic users** |
| --- | --- |
| Overall cohort | 143 (29.3) |
| Esophagus | 5 (31.3) |
| Stomach | 54 (39.7) |
| Colorectum | 4 (44.4) |
| Kidney | 22 (34.4) |
| Lung | 53 (26.0) |
| **Organ of the primary tumor** | **CBM588 users** |
| Overall cohort | 89 (20.5) |
| Esophagus | 4 (26.7) |
| Stomach | 39 (32.2) |
| Colorectum | 4 (44.4) |
| Kidney | 14 (25.0) |
| Lung | 32 (17.5) |

**Table R3. Overall survival treated with nivolumab according to probiotics use for several cancers**

| **Organ of the primary tumor** | **Any probiotic user**  **(N = 143)** | **Probiotic non-user**  **(N = 345)** | **HR (95% CI)** | ***p*** |
| --- | --- | --- | --- | --- |
| All | 702.5 | 470.0 | 0.68 (0.47–0.99) | **0.042*** |
| Esophagus (n = 16) | 1596.0 | 657.0 | - | - |
| Stomach (n = 136) | 690.0 | 443.0 | 0.54 (0.29–1.01) | 0.052 |
| Colorectum (n = 9) | 909.0 | 398.0 | - | - |
| Kidney (n = 64) | 437.5 | 296.0 | 0.39 (0.11–1.41) | 0.151 |
| Lung (n = 204) | 746.0 | 552.0 | 0.82 (0.49–1.37) | 0.443 |
| **Organ of the primary tumor** | **CBM588 user**  **(N = 89)** | **Probiotic non-user**  **(N = 345)** | **HR (95% CI)** | ***P*** |
| All | 690.0 | 470.0 | 0.69 (0.45–1.08) | 0.102 |
| Esophagus (n = 15) | 1266.0 | 657.0 | - | - |
| Stomach (n = 121) | 691.0 | 443.0 | 0.46 (0.22–0.95) | **0.036*** |
| Colorectum (n = 9) | 909.0 | 398.0 | - | - |
| Kidney (n = 56) | 458.5 | 296.0 | 0.21 (0.026–1.60) | 0.130 |
| Lung (n = 183) | 718.0 | 552.0 | 1.00 (0.55–1.80) | 0.997 |

*Shows <0.05.

Abbreviations: HR, hazard ratio; CI, confidence interval.

**Supplementary Figure 1. Cumulative incidence of nivolumab continuation in probiotic users vs non-users for (A) esophageal cancer, (B) colorectal cancer, (C) renal cancer,** **and (D) lung cancer**


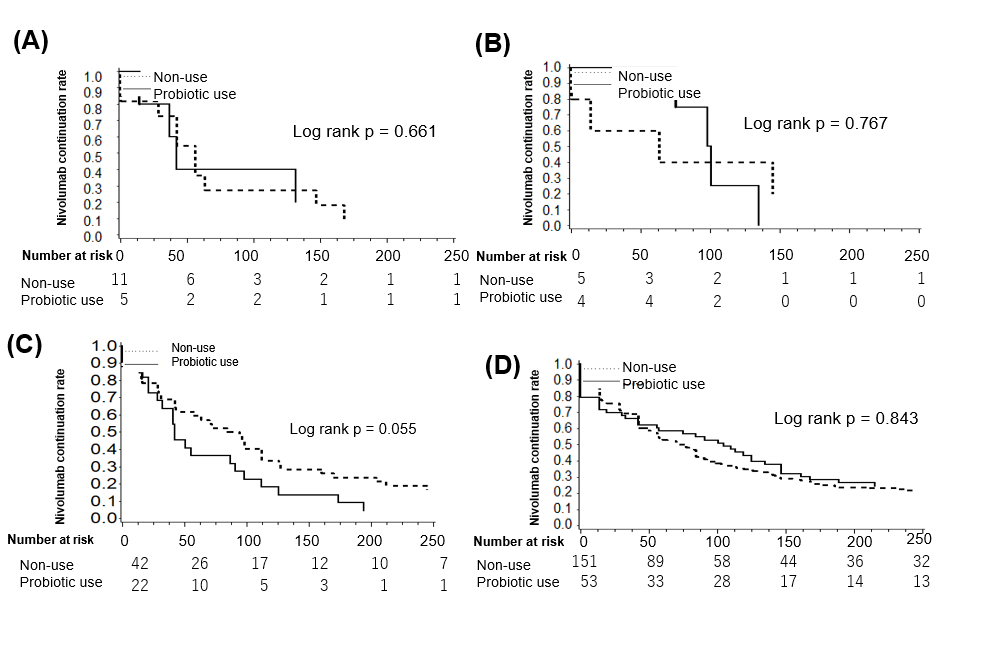

Supplement: Supplementary file 1 — Data S1: [file CAM4-12-16876-s001.docx]
